# Unpaid care, time taken off work and healthcare costs before and after partner bereavement among same-gender and different-gender partners: A national population-based study

**DOI:** 10.1177/02692163251355796

**Published:** 2025-08-16

**Authors:** Katherine Bristowe, Peter May, Alexandra Pitman, Jingjing Jiang, Liadh Timmins, Michael King, Debbie Braybrook, Steve Marshall, Elizabeth Day, Paul Clift, Ruth Rose, Katherine Johnson, Kathryn Almack, Richard Harding

**Affiliations:** 1 Cicely Saunders Institute of Palliative Care, Policy & Rehabilitation, Florence Nightingale Faculty of Nursing Midwifery and Palliative Care, King’s College London, London, UK; 2 School of Medicine, Trinity College Dublin, Dublin, Ireland; 3 UCL Division of Psychiatry, UCL (University College London), London, UK; 4 North London NHS Foundation Trust, London, UK; 5 Queen Mary University of London, London, UK; 6 School of Psychology, Swansea University, Swansea, UK; 7 King’s College Hospital, London, UK; 8 Patient & Public Involvement Member, UK; 9 RMIT, Melbourne, VIC, Australia; 10 University of Hertfordshire, Hatfield, UK

**Keywords:** Bereavement, sexual minority, sexual orientation, health service utilisation, health care costs, labour force, informal care

## Abstract

**Background:**

Recent research has demonstrated higher levels of psychological distress for bereaved same-gender partners compared to different-gender partners. Economic outcomes have not yet been examined.

**Aim:**

To examine whether there are differences between same- and different-gender civil partners or spouses (hereafter ‘partners’) in the amount of unpaid care provided in the 3 months pre-bereavement, and time taken off work and formal healthcare used in the 3 months pre- or post-bereavement.

**Design:**

A population-based cross-sectional survey of bereaved partners from England/Wales was conducted including three economic outcomes of interest: unpaid care, time taken off work, and formal healthcare used. We estimated formal healthcare costs using reference costs. We balanced groups on sociodemographic characteristics using propensity score weights and estimated average marginal difference in outcomes between groups using multivariable regressions.

**Setting/participants:**

There were 542 complete cases for primary analysis (220 same-gender partners, 322 different-gender partners).

**Results:**

Same- and different-gender partners provided very high levels of unpaid care pre-bereavement (mean 122 h/week). Of those in paid employment, 85% missed some work pre- and post-bereavement. Same-gender partners had higher formal healthcare costs post-bereavement (+£79, 95% CI: +2 to +156). There were no other significant differences between groups.

**Conclusion:**

The economic burdens of bereavement are substantial. Same-gender partners were associated with more formal healthcare use than different-gender partners post-bereavement, possibly connected to higher levels of psychological distress. Future research should consider longer-term impacts of partner bereavement on health outcomes, explore whether care services are experienced as inclusive, and target ethnically diverse and gender diverse communities.

**What is already known about the topic?**
  - Bereaved partners experience high levels of grief intensity in bereavement irrespective of their gender concordance (same-gender partner or different-gender partner).
  - Bereaved same-gender partners experience significantly higher levels of psychological distress in bereavement compared to different-gender partners.
  - Bereavement is associated with significant economic burdens, including provision of unpaid care and time taken off work, however inequities in these burdens are less well described.
**What this paper adds?**
  - High levels of unpaid informal care were provided by bereaved partners in the last three months of their partner’s life, irrespective of gender concordance.
  - Of those partners in paid employment, 85% missed days of work to provide care for their partner in the last three months of their life.
  - Same-gender partners had higher healthcare costs after the death of their partner than different-gender partners.
**Implications for practice, theory or policy**
  - It is essential that the high levels of caregiving provided by partners and significant others are acknowledged when considering policy and service reform.
  - Health and social care services need to consider how best to meet the needs of this growing population of unpaid caregivers.
  - Those bereaved of a same gender partner in our sample used more healthcare services in bereavement, suggestive of a cumulative and reinforcing effect of discrimination, worse general mental health and the impact of bereavement.

## Introduction

### Background

Approximately 600,000 adults die in the UK each year,^1^ and over 60 million people globally.^2^ Those closest to the deceased experience significantly worse health outcomes pre- and post-bereavement than non-bereaved controls,^3^ are less likely to be in paid employment 2 years later,^4^ and experience higher rates of mortality and hospitalisation.^3^ Bereaved partners are more likely than non-bereaved partners to develop new health problems or experience a recurrence of an existing problem in the year following the bereavement, but less likely to access care when needed.^5^ After bereavement 10%–20% of people experience a prolonged grief reaction, resulting in difficulties returning to usual activities, necessitating professional bereavement or psychological support.^6^

Around 1.5 million adults identified themselves as lesbian, gay or bisexual in England and Wales in the 2021 Census (3.2% of the population).^7^ However, a recent global survey of 30 countries suggests estimates of people with a minoritised sexual orientation may be as high as 8%.^8^ Lesbian, gay, bisexual and trans (LGBT+) people have higher prevalence of certain serious physical illnesses,^9^ mental illness, substance misuse and suicidal thoughts than heterosexual people,^10^ thought to be linked to the discrimination they experience.^11^ UK healthcare organisations have a legal duty to reduce inequalities in access to care services for minoritised groups.^12^ Despite the legislative change to support the rights of LGBT+ people,^13^ experiences and fears of discrimination persist in health and social care.^14–17^ Globally, although some advances in equality are evident for LGBT+ people, non-governmental organisations have reported that, as of 2024, 65 countries criminalise same-sex sexual activity, of which 12 can impose the death penalty.^18,19^

Population-based studies investigating the mental health of LGBT+ communities have been conducted,^10,11^ but such studies have not been applied to bereavement. A systematic review of bereavement outcomes and experiences of LGBT+ bereaved partners^15^ identified qualitative evidence of additional barriers and stressors for bereaved LGBT+ partners, but a total absence of quantitative studies into bereavement outcomes of LGBT+ partners since the 1990s and beyond the context of HIV/AIDS. Our recent population-based comparative study of outcomes in bereavement for same-gender and different-gender partners was the first of its kind in the context of bereavement.^20^ It demonstrated high levels of grief intensity across both groups, but significantly higher psychological distress amongst same-gender partners. It found evidence to support loneliness as a potential mediator of the association between same-gender versus different-gender partner bereavement and grief intensity, and a similar potential role for social support, loneliness and caregiver burden in the association between partner gender concordance and psychiatric symptoms. These suggest potentially important relationships between the caregiving role, support networks and wellbeing for bereaved partners.

### Rationale and aims

Although life expectancy is increasing in many societies, many people experience poor health in older age, and need help with activities of daily living, with much of this caregiving undertaken by partners and family.^21^ One important evidence gap in relation to bereavement experiences of LGBT+ communities relates to health service utilisation and costs. Formal healthcare costs increase near end-of-life^22^ and unpaid care costs are of a similar magnitude to formal costs.^23^ Providing informal care imposes adverse health and social effects on the carer, with increased risk of hospitalisation and of leaving education or employment.^24,25^ The burdens of unpaid care often fall inequitably with respect to diagnosis, socio-economic status, gender, ethnicity and employment status.^26^ We are unaware of any previous studies comparing these outcomes between same-gender partners and different-gender partners in the bereavement context.

In this paper we analysed economic data from our population-based survey to address three research questions: (1) Were there differences in the amount of unpaid care provided by same-gender and different gender partners in the final 3 months of the deceased partner’s life? (2) Were there differences in time taken off work among same-gender and different gender partners in the 3 months pre- and post-bereavement? (3) Were there differences in formal healthcare costs among same-gender and different-gender partners in the 3 months pre- and post-bereavement?

## Methods

### Study design

This analysis forms part of a population-based cross-sectional mixed-methods (retrospective post-bereavement survey and in-depth qualitative interviews) study of bereavement outcomes and experiences for same gender and different gender bereaved civil partners or spouses (hereafter ‘partners’).

### Population

We analysed primary data from a population-based cross-sectional survey of bereaved partners or spouses in England and Wales.^20^

### Sampling

The UK Office for National Statistics (ONS) conducted sampling on our behalf to protect anonymity and sent out invitations based on death registration data. Individuals who registered the death of a same-gender or different-gender civil partner or spouse were identified by relationship (wife, widow, husband, widower, civil partner) and gender of decedent (male, female) in the death registry data.

### Recruitment

Invitations were sent to 564 individuals who had consecutively registered the death of a same-gender partner (between 9 September 2017 and 8 January 2019), and a random sample of 1380 individuals who had registered the death of a different-gender partner during the same period. Invitations were sent 6–10 months post bereavement to avoid the immediate bereavement period and the anniversary of the death, as each are a marker for heightened grief.^27,28^ Survey packs included a paper copy of the questionnaire (see Supplemental Materials), an opt-out form, and bereavement support literature, as well as web links for online copies of the questionnaire and opt-out form. A single reminder was sent 2–3 weeks after the initial invitation to invitees who had not yet responded. We included questions about gender and sexual orientation of the participants in the questionnaire (with an option of ‘prefer not to say’) to ascertain the relationship between the deceased and the participant. Participants consented to the study by returning the completed paper or online survey.

### Variables

#### Dependent variables

For our first research question on unpaid care, bereaved partners were asked how much care was provided in the last 3 months of life with regard to six types of care: personal care (e.g. washing, dressing); medical procedures (e.g. taking medicines); going to appointments or treatments; household tasks; time spent together; and time spent ‘on call’. Available responses were categorical for informal input (<5 h, 5–9 h, 10–19 h, 20–49 h, 50⩽ h). To estimate total hours per week we summed the mid-point of each categorical response. In the context of over-reporting of similar data,^29^ we imposed a maximum of 16 h per day for *active* care (personal care, medical procedures, going to appointments, household tasks) and a maximum of 24 h per day for all care (active care plus time spent together and time spent on call). Since the data were collected categorically, such that the derived ‘total care hours’ variable was not truly continuous, in analysis we expressed total care hours as a categorical variable: less than 6 h per day; 6–16 h; more than 16 h.

For our second research question on time taken off work, bereaved partners were asked if they were in paid employment and, if so, if they had taken any time off work in the 3 months pre- and post-bereavement, operationalised as a binary variable. For those who had missed work, we asked how many days of work were missed (integer value; continuous variable). Due to the high degree of missing data for this continuous variable we used only the binary variable in our models.

For our third research question on healthcare costs in the 3 months pre- and post-bereavement, bereaved partners were asked about frequency of healthcare utilisation using a version of the Client Service Receipt Inventory (CSRI), a service utilisation tool that is commonly restricted to services relevant to the population of interest.^30^ We restricted this to use of formal healthcare, then identified unit costs for each service standardised to 2023 values using established sources for such costs,^31,32^ and estimated the costs of care by combining unit costs with reported frequencies.

#### Independent variables

In all three analyses our primary independent variable was binary: same-gender bereaved partner or different-gender bereaved partner to the deceased. Other predictors were selected based on hypothesised connection with outcome, or primary independent variable and outcome.

Predictors that we considered suitable for all analyses were: age, gender, race and ethnicity and religiosity of both the bereaved and the deceased partners; and; education of the bereaved partner; life circumstances of the bereaved (employment, proximity to nearest relative), experience of discrimination in health and social care (Everyday Discrimination Scale^33^), and whether or not the death was expected based on empirical cut-offs^34^ (See Table 1).

**Table 1.** Participant characteristics (*N* = 542), after propensity score weighting.

Participant characteristics	*n* = 322	*n* = 220	*n* = 542
	Different-gender partners	Same-gender partners	All
	Mean (SD or *n*)	Mean (SD or *n*)	Mean (SD or *n*)
*Deceased partner*			
Age: *Years (SD)*	68 (13)	68 (13)	68 (13)
Gender: *Female*	47% (150)	35% (76)	41% (226)
*Male*	53% (172)	65% (144)	59% (316)
Race/ethnicity: *Minority ethnic*	2% (7)	3% (7)	3% (14)
Religion: *Yes*	76% (246)	62% (136)	69% (382)
*Bereaved partner*			
Age: *Years (SD)*	66 (13)	64 (11)	65 (12)
Gender: *Female*	53% (172)	35% (76)	44% (248)
*Male*	47% (150)	65% (144)	56% (294)
Race/ethnicity: *Minority ethnic*	2% (8)	3% (7)	3% (15)
Religion: *Yes*	72% (233)	57% (125)	65% (358)
Education: *University*	39% (125)	45% (99)	42% (224)
Employment: *Paid employment*	37% (118)	31% (69)	34% (187)
*Retired*	54% (175)	53% (116)	54% (291)
*Neither*	9% (29)	16% (35)	12% (64)
Help nearby: *Yes*	78% (250)	67% (147)	72% (397)
Discrimination: *Yes*	10% (33)	12% (26)	11% (59)
Unexpected death: *Yes*	22% (71)	20% (44)	21% (115)

Help nearby: About how long does it take for your nearest relative or friend to get to where you live? (recoded from seven levels as a binary—more or less than 30 min). Discrimination: In your day-to-day life, how often do you feel you are treated unfairly by health and social care professionals? (recoded from six levels as a binary, Never = No; Ever = Yes). Unexpected death: Did the bereaved person first understand their partner was dying less than an hour before death, or not until after death? SD: standard deviation reported for continuous variables; cell size (*n*) reported for categorical variables.

### Statistical methods

#### Sample size and missing data

In devising the original survey, we had based our sample size calculation on a primary outcome measure (complicated grief) for bereavement outcomes, as reported in a previous analysis.^20^ The survey response rate was 29.3% (569/1945).

For research questions 1 and 3 in the present analysis, respondents were excluded if they were missing data on outcomes (unpaid care provided; healthcare costs), age, gender, race and ethnicity or education. Where participants were missing data on other predictors, we imputed the median.

For research question 2 we restricted this sample to those in paid work who answered the binary outcome variable (did they or did they not take any time off work).

#### Handling missing data

The same-gender partner and different-gender partner groups differed on variables collected in our data, including age and gender (see Supplemental Table 1), and were likely to differ on unmeasured variables.^35^ As missing data on observed confounders and failure to adjust for unobserved confounders could bias our estimates of association between our primary independent variable and our outcomes,^35^ we examined the data for an instrumental variable that would allow us to control for both observed and unobserved confounding.^36^ However, we were unable to identify a valid instrument. We therefore used propensity scores to control for differences between groups on observed characteristics only.^37^ We employed inverse probability of treatment weighting using sociodemographic and life experience variables. We recalculated the propensity score for research question 2, which involved a different sampling frame than questions 1 and 3.

#### Regression models

For question 1 we used an ordered probit regression with a categorical outcome variable for volume of unpaid care provided. For research question 2 we used logistic regressions for a binary outcome. For research question 3 we used a generalised linear model (GLM) with a gamma distribution and a log link, selected at the same time based on information criteria in model diagnosis.^38^

For primary analysis in questions 2 and 3 we evaluated the outcome for the entire 6-month period covered by data collection. In secondary analysis we evaluated each outcome separately for the 3 months preceding the death and the 3 months following.

In all analyses, predictors were those listed in Table 1. For all analyses we report only the estimated marginal effect for the primary independent variable. For discrete outcome variables, this marginal effect quantifies how the predicted probabilities change as the predictor increases from 0 to 1. For continuous variables, the marginal effect is the estimated change in outcome as the predictor increases from 0 to 1. This choice reflects the relative strengths of marginal effects for interpretation and generalisability to other studies,^39^ and to minimise the risk of ‘Table 2 fallacies’, which are heightened when propensity score weights are applied.^40^

**Table 2.** Economic outcomes of interest.

Economic outcomes	*n* = 322	*n* = 220	*n* = 542
	Different-gender partners	Same-gender partners	All
	Mean (SD or *n*)	Mean (SD or *n*)	Mean (SD or *n*)
*Question 1*			
Unpaid care hours (total per week)	118.2 (51.4)	124.9 (46.3)	121.5 (49.0)
hrs per day ⩽6	11% (30)	8% (21)	9% (51)
6< h per day ⩽16	32% (86)	28% (76)	30% (162)
16< h per day ⩽24	57% (156)	64% (174)	61% (329)
*Question 2*			
In paid employment Yes (*n* = 547)	27% (87)	40% (88)	32% (175)
Missed work to provide care (*n* = 175)	80% (70)	90% (79)	85% (149)
Missed work before bereavement (*n* = 175)	70% (59)	81% (70)	76% (129)
Missed work after bereavement (*n* = 175)	82% (71)	89% (78)	85% (149)
Total days missed work ( *n* = 60^a^)	74.0 (44.1)	68.5 (54.5)	70.8 (49.4)
*Question 3*			
Total formal costs, 3 months prior	£728 (1460)	£696 (1079)	£712 (1283)
Total formal costs, 3 months after	£201 (330)	£279 (449)	£240 (396)
Total formal costs, total	£929 (1537)	£974 (1253)	£952 (1401)

a Of the 149 people to report missing work, 60 (40%) responded to the question about days missed. SD: standard deviation reported for continuous variables; cell size (*n*) reported for categorical variables.

#### Sensitivity analyses

We reran our main analyses without additional model predictors (to check if imputation of missing values among some predictors was biasing results), without propensity score weighting (to check if the weights were biasing results) and using a different regression model for costs (to check that model choice did not drive results).

#### Software

All analyses were performed using Stata version 17.^41^

## Results

### Descriptive data

There were 561 respondents to the survey^20^ of whom 542 had sufficient data for this analysis. The key characteristics of the deceased and bereaved participants are presented in Table 1, following propensity score weighting. For the unweighted data and details of those excluded due to missingness see Supplemental Materials.

There were 220 (41%) same-gender partners and 322 (59%) different-gender partners, with an average age of 64 and 66 years respectively. In the weighted sample, females were a minority both among the deceased partners (41%) and the bereaved partners (44%). An approximately two-thirds majority in both groups identified as having a religion. The most common employment situation among bereaved partners was retired (54%). Most bereaved partners (72%) had another family relative living within half an hour, and a minority of deaths among partners (21%) were unexpected. A minority of bereaved partners (11%) reported experiencing discrimination in receiving health and social care.

### Outcome data

The mean hours of unpaid care per week during the last 3 months of life was 122, with a majority (61%) of bereaved people reporting more than 16 h per day (Table 2). One third (*n* = 175) of the bereaved partners were in paid employment, of whom 149 (85%) reported missing work at some point in the 3 months before or after the death. Only 60 (40%) of those in work specified how many days they had missed, with an average of 71 (out of a possible 130, assuming a 5-day working week for 13 weeks before death and 13 weeks after death).

### Main results

**Question 1: Group differences in provision of unpaid care by bereaved partners in the 3 months prior to death**

There was a positive association between same-gender partners and higher volume of unpaid care, but none of the relationships were statistically significant (Table 3).

**Table 3.** Association between characteristics and unpaid care hours (*n* = 542).

Relationship: *Same gender*	Marginal effect	95% Confidence interval
Hours per day ⩽6	−0.02	−0.06 to 0.01
6< h per day ⩽16	−0.04	−0.11 to 0.02
16< h per day ⩽24	0.07	−0.02 to 0.16

**Question 2: Group differences in bereaved partners taking any time off work**

There was a positive association between same-gender partners taking any time off work, but none of the relationships were statistically significant (Table 4).

**Table 4.** Association between characteristics and time taken off work (*n* = 175).

Relationship: *Same gender*	Marginal effect	95% Confidence interval
Missed work: Any time	0.07	−0.04 to 0.19
Missed work: Before bereavement	0.07	−0.06 to 0.21
Missed work: When bereaved	0.02	−0.08 to 0.12

**Question 3: Group differences in formal healthcare costs prior to and after the death**

There was a positive association between same-gender partners and healthcare costs for the 6 months that spanned the bereavement, and a negative association between same-gender partners costs before the death, but neither was statistically significant (Table 5). There was a statistically significant positive association between same-gender partners and healthcare costs after the death (+£79, 95% CI: 2–156).

**Table 5.** Association between characteristics and healthcare costs (*n* = 542).

Relationship: *Same gender*	Marginal effect (£)	95% confidence interval
Healthcare costs: All 6 months	45	−270 to 360
Healthcare costs: 3 months prior	−33	−331 to 265
Healthcare costs: 3 months after	79	2 to 156

### Sensitivity analyses

We reran our main analyses without additional model predictors (to check if imputation of missing values among some predictors was biasing results), without propensity score weighting (to check if the weights were biasing results) and using a different regression model for costs (to check that model choice did not drive results).

The positive association between same-gender partners and healthcare costs in bereavement remained statistically significant, and all other relationships remained statistically non-significant, that is, all results reported for our main analyses were robust to the checks undertaken in our sensitivity analyses (see Supplemental Materials).

## Discussion

### Key results

Our national population-based survey of bereaved partners found that high levels (mean 122 h/week) of unpaid informal care were provided by bereaved partners in the last 3 months of their partner’s life, regardless of gender concordance (same-gender or different-gender partnership). Of those partners in paid employment, 85% missed days of work to provide care for their partner during this time. There was a statistically significant association between same-gender partners and higher healthcare costs after the death of their partner, possibly linked to their significantly higher levels of psychological distress.^20^

### Findings in the context of other studies

More people are living into older age, but many are living with illnesses or disability, creating additional day-to-day challenges. It is estimated that 48% more of people over 65 years in England will require help day-to-day by 2038 (increasing from 3.5 million to 5.2 million).^21^ This represents high levels of caregiving contribution made by partners and significant others, which must be taken into account to inform adequate policy and service reform.

Another important consideration alongside the impact of caregiving for surviving partners is the impact of loneliness and inadequate social support on the mental health of bereaved partners.^20,42^ It is recognised that, compared to non-bereaved people, those who have experienced a bereavement have significantly worse health outcomes both pre- and post-bereavement, and experience higher rates of mortality and hospitalisation.^3^ Following partner bereavement, individuals are also less likely to access health and social care when they need it.^5^ In the context of the projected increase in care needs, this could result in rising unaddressed or unmet needs. Those bereaved of a same-gender partner in our sample used significantly more healthcare services in bereavement. Alongside the higher psychological distress experienced by bereaved same-gender partners compared to different-gender partners,^20^ this is suggestive of a cumulative and reinforcing effect of discrimination, worse general mental health and the impact of bereavement. Indeed, a recent study in the UK found that more than two thirds of LGBT+ people would avoid holding hands with a same-sex partner for fear of a negative reaction, and two-fifths had experienced an incident of harassment or violence in the past year.^43^ This hostile environment for LGBT+ people, and the persistent fear of discrimination, is likely to contribute to poorer mental health, and a reduced satisfaction with life compared to the UK general population.^43^

### Strengths and limitations

The methods used in this study, and the rigour with which it was undertaken, (i.e. the population-based sampling) advance the science of research with LGBT+ communities. Population-based studies with LGBT+ communities are still relatively rare, and as such successfully applying this approach underscores the originality and significance of this work. The data on lost working days by informal caregivers pre-death and into bereavement offer important new insights for policy makers. However, this work had limitations. The cross-sectional design of the study limits our ability to explore outcomes and service utilisation throughout the trajectories of bereavement, as well as to generate credible causal estimates of relationship between dependent variables and primary independent variable. Each of our reported marginal effect estimates had substantial associated uncertainty. We controlled for material well-being directly through employment status and indirectly through education, but there remain unobserved confounders in this domain such as income and assets. Our measure of time taken off work was relatively crude, as it related to number of full days taken off paid work. This underestimates lost days of productivity for those who work freelance, or partial days lost. The low response rate on number of lost days raises potential concerns over representativeness of descriptive data as this high missingness is likely not at random. Further, this missingness prevented us from analysing the lost days data in the main analyses; we instead analysed only binary variables of whether work days were lost or not. Also, whilst we are able to examine service utilisation, we were unable to explore the extent to which those services were inclusive. Importantly, in this survey there were low levels of participation (3%) from people from minoritised ethnic groups. It is possible that individuals from minoritised ethnic groups were less likely to be married or civil partnered, or to register the death of the partner, which would have excluded them from our recruitment processes. Non-response bias is also possible in the context of experiences or fears of discrimination which may have precluded their participation. A small number of participants (*n* = 16) in the study identified as bisexual, and fewer as transgender (*n* < 10). Due to small numbers, it was not possible to explore whether bisexuality or gender modality influenced outcomes, which further contributes to the invisibility of these groups in research.

### Conclusions

This study demonstrates that, irrespective of gender concordance, bereaved partners provide high levels of informal care pre-bereavement and most partners in paid work miss substantial numbers of days of work to provide care. It is essential that the high levels of caregiving provided by partners and significant others are acknowledged when considering policy and service reform. Moreover, further work is needed to examine the longer-term impact of caregiving and partner bereavement on health outcomes in order to understand how best to meet the needs of this growing population of unpaid caregivers. Being in a same-gender relationship was associated with higher health service utilisation post-bereavement, which may relate to previous findings about higher levels of psychological distress among those in same-gender relationships after partner loss, and may be suggestive of a cumulative and reinforcing effect of discrimination, worse general mental health and the impact of bereavement. Future work should explore and validate this association using other data and analytic methods. Further research should also examine the extent to which health and care services were perceived to be inclusive, and target ethnically diverse and gender diverse communities.

## Supplemental Material

sj-docx-1-pmj-10.1177_02692163251355796 – Supplemental material for Unpaid care, time taken off work and healthcare costs before and after partner bereavement among same-gender and different-gender partners: A national population-based study Supplemental material, sj-docx-1-pmj-10.1177_02692163251355796 for Unpaid care, time taken off work and healthcare costs before and after partner bereavement among same-gender and different-gender partners: A national population-based study by Katherine Bristowe, Peter May, Alexandra Pitman, Jingjing Jiang, Liadh Timmins, Michael King, Debbie Braybrook, Steve Marshall, Elizabeth Day, Paul Clift, Ruth Rose, Katherine Johnson, Kathryn Almack and Richard Harding in Palliative Medicine

sj-docx-2-pmj-10.1177_02692163251355796 – Supplemental material for Unpaid care, time taken off work and healthcare costs before and after partner bereavement among same-gender and different-gender partners: A national population-based study Supplemental material, sj-docx-2-pmj-10.1177_02692163251355796 for Unpaid care, time taken off work and healthcare costs before and after partner bereavement among same-gender and different-gender partners: A national population-based study by Katherine Bristowe, Peter May, Alexandra Pitman, Jingjing Jiang, Liadh Timmins, Michael King, Debbie Braybrook, Steve Marshall, Elizabeth Day, Paul Clift, Ruth Rose, Katherine Johnson, Kathryn Almack and Richard Harding in Palliative Medicine
