# Supplementary material for: Unpaid care, time taken off work and healthcare costs before and after partner bereavement among same-gender and different-gender partners: A national population-based study
Source: Palliat Med. 2025 Aug 16;39(9):977–86. doi: 10.1177/02692163251355796 (PMC12476458; doi:10.1177/02692163251355796)
Supplement: sj-docx-1-pmj-10.1177_02692163251355796 – Supplemental material for Unpaid care, time taken off work and healthcare costs before and after partner bereavement among same-gender and different-gender partners: A national population-based study [file sj-docx-1-pmj-10.1177_02692163251355796.docx]

# Appendix

## Comparison of samples

#### Included versus excluded

In Appendix Table 1 below we present the Table 1 data for those participants included in the main paper (N=542), and those excluded due to missing baseline variables (n=26). Missingness was most common in sociodemographics variables (Age, Gender, minoritised ethnic group).

We calculated absolute standardised differences (ASD), a measure of variation between groups that does not rely on sample size, and where 25%<ASD is considered a potentially concerning magnitude of difference.^1^ Mean ASD across all Table 1 variables was 12% and only two variables exceeded the 25% threshold, i.e. we considered that those excluded from the sample did not differ substantively from those retained.

#### Effect of propensity score weights

In Appendix Table 2 below we present the Table 1 data for those participants included in questions 1 and 3 in the main paper (N=542) with and without propensity score weights. Mean ASD prior to weighting was 28% and 7 of 15 variables breached the threshold, i.e. there was considerable variation. In particular in the raw sample there were large differences in age, gender and university education.

Following weighting the mean ASD was 13% and three variables breached the threshold. Therefore, the weights had a marked effect on improving balance between the groups, and we considered the residual differences in weighted data to be reasonable in context.

Equivalent balance was achieved in the sub-sample for question 2, which included people in employment only [data not shown].

Appendix Table 1 Comparison of those included in the sample and excluded due to missing data

|  |  | **EXCLUDED** | | | | | | | **INCLUDED** | | | | | |  |
| --- | --- | --- | --- | --- | --- | --- | --- | --- | --- | --- | --- | --- | --- | --- | --- |
|  |  |  | **Different Gender** | | **Same Gender** | | **ALL** | | **Different Gender** | | **Same Gender** | | **ALL** | |  |
|  |  | **n_ans_** | **n= 14** | | **n=12** | | **N= 26** | | **n= 322** | | **n= 220** | | **N= 542** | |  |
| **Deceased partner** | |  | **Mean** | **SD** | **Mean** | **SD** | **Mean** | **SD** | **Mean** | **SD** | **Mean** | **SD** | **Mean** | **SD** | **ASD** |
| **Age** | *Years (SD)* | 20 | 73 | 9 | 70 | 15 | 71 | 12 | 73 | 11 | 68 | 13 | 71 | 12 | <0.5% |
| **Gender** | *Female (n)* | 23 | 0.64 |  | 0.92 |  | 0.78 |  | 0.47 |  | 0.35 |  | 0.41 |  | 11% |
|  | *Male (n)* |  | 0.36 |  | 0.08 |  | 0.22 |  | 0.53 |  | 0.65 |  | 0.59 |  |  |
| **Minoritised ethnic group** |  | 17 | 0.13 |  | 0.00 |  | 0.06 |  | 0.02 |  | 0.03 |  | 0.03 |  | 12% |
| **Religious** |  | 26 | 0.71 |  | 0.67 |  | 0.69 |  | 0.80 |  | 0.62 |  | 0.73 |  | 6% |
| **Bereaved partner** | |  |  |  |  |  |  |  |  |  |  |  |  |  |  |
| **Age** | *Years (SD)* | 21 | 74 | 8 | 61 | 16 | 67 | 14 | 70 | 11 | 64 | 11 | 68 | 11 | 8% |
| **Gender** | *Female (n)* | 22 | 0.36 |  | 0.92 |  | 0.61 |  | 0.53 |  | 0.35 |  | 0.44 |  | 40% |
|  | *Male (n)* |  | 0.64 |  | 0.08 |  | 0.39 |  | 0.47 |  | 0.65 |  | 0.56 |  |  |
| **Minoritised ethnic group** |  | 10 | 0.00 |  | 0.00 |  | 0.00 |  | 0.02 |  | 0.03 |  | 0.03 |  | 24% |
| **Religious** |  | 26 | 0.71 |  | 0.67 |  | 0.69 |  | 0.80 |  | 0.57 |  | 0.71 |  | 3% |
| **Education** | *University* | 26 | 0.21 |  | 0.58 |  | 0.38 |  | 0.20 |  | 0.45 |  | 0.30 |  | 14% |
| **Employ.** | *Paid employ.* | 25 | 0.00 |  | 0.50 |  | 0.24 |  | 0.24 |  | 0.31 |  | 0.27 |  | 6% |
|  | *Retired* | 25 | 1.00 |  | 0.33 |  | 0.68 |  | 0.68 |  | 0.53 |  | 0.62 |  | 10% |
|  | *Neither* | 25 | 0.00 |  | 0.17 |  | 0.08 |  | 0.07 |  | 0.16 |  | 0.11 |  | 8% |
| **Help nearby** | *mins<30* | 23 | 0.75 |  | 0.36 |  | 0.57 |  | 0.79 |  | 0.67 |  | 0.74 |  | 30% |
| **Experienced discrimination** | | 25 | 0.08 |  | 0.17 |  | 0.12 |  | 0.12 |  | 0.12 |  | 0.12 |  | <0.5% |
| **Unexpected death** | | 26 | 0.21 |  | 0.17 |  | 0.19 |  | 0.20 |  | 0.20 |  | 0.20 |  | 2% |
|  | | | | | | | | | | | | | MEAN ASD= | | 12% |
| For variable definitions, see Table 1 in main paper. n_ans_= number of excluded participants who answered a specific question. ASD: absolute standardised difference between ALL excluded and ALL included, high values (25%<) marked in red. | | | | | | | | | | | | | | | |

Appendix Table 2 Comparison of analytic sample with and without propensity score weights

|  |  | **WEIGHTED, PER TABLE 1 IN MAIN PAPER** | | | | | | | **UNWEIGHTED** | | | | | |  |  |
| --- | --- | --- | --- | --- | --- | --- | --- | --- | --- | --- | --- | --- | --- | --- | --- | --- |
|  |  |  | **Different Gender** | | **Same Gender** | | **ALL** | | **Different Gender** | | **Same Gender** | | **ALL** | |  |  |
| **Deceased partner** | |  | **Mean** | **SD** | **Mean** | **SD** | **Mean** | **SD** | **Mean** | **SD** | **Mean** | **SD** | **Mean** | **SD** | **ASD_W_** | **ASD_U_** |
| **Age** | *Years (SD)* |  | 68 | 13 | 68 | 13 | 68 | 13 | 73 | 11 | 68 | 13 | 71 | 12 | <0.5% | 42% |
| **Gender** | *Female (n)* |  | 47% |  | 35% |  | 41% |  | 23% |  | 35% |  | 27% |  | 20% | 22% |
|  | *Male (n)* |  | 53% |  | 65% |  | 59% |  | 77% |  | 65% |  | 73% |  |  |  |
| **Minoritised ethnic group** |  |  | 2% |  | 3% |  | 3% |  | 2% |  | 3% |  | 3% |  | 5% | 4% |
| **Religious** |  |  | 76% |  | 62% |  | 69% |  | 80% |  | 62% |  | 73% |  | 26% | 34% |
| **Bereaved partner** | |  |  |  |  |  |  |  |  |  |  |  |  |  |  |  |
| **Age** | *Years (SD)* |  | 66 | 13 | 64 | 11 | 65 | 12 | 70 | 11 | 64 | 11 | 68 |  | 17% | 55% |
| **Gender** | *Female (n)* |  | 53% |  | 35% |  | 44% |  | 77% |  | 35% |  | 60% |  | 30% | 80% |
|  | *Male (n)* |  | 47% |  | 65% |  | 56% |  | 23% |  | 65% |  | 40% |  |  |  |
| **Minoritised ethnic group** |  |  | 2% |  | 3% |  | 3% |  | 2% |  | 3% |  | 3% |  | 5% | 4% |
| **Religious** |  |  | 72% |  | 57% |  | 65% |  | 80% |  | 57% |  | 71% |  | 26% | 44% |
| **Education** | *University* |  | 39% |  | 45% |  | 42% |  | 20% |  | 45% |  | 30% |  | 10% | 46% |
| **Employ.** | *Paid employ.* |  | 37% |  | 31% |  | 34% |  | 24% |  | 31% |  | 27% |  | 10% | 13% |
|  | *Retired* |  | 54% |  | 53% |  | 54% |  | 68% |  | 53% |  | 62% |  | 2% | 27% |
|  | *Neither* |  | 9% |  | 16% |  | 12% |  | 7% |  | 16% |  | 11% |  | 18% | 23% |
| **Help nearby** | *mins<30* |  | 78% |  | 67% |  | 72% |  | 79% |  | 67% |  | 74% |  | 21% | 24% |
| **Experienced discrimination** | |  | 10% |  | 12% |  | 11% |  | 12% |  | 12% |  | 12% |  | 5% | 1% |
| **Unexpected death** | |  | 22% |  | 20% |  | 21% |  | 20% |  | 20% |  | 20% |  | 4% | <0.5% |
|  | | | | | | | | | | | | | MEAN ASD= | | 13% | 28% |
| For variable definitions, see Table 1 in main paper. ASD: absolute standardised difference, high values (25%<) marked in red. ASD_W_ presents the differences in the weighted sample – what is the ASD between different-gender groups and same-gender group for each variable? ASD_U_ presents the differences in the unweighted sample. A comparison of ASD_W_ and ASD_U_ therefore quantifies the balancing effect of the weights on observed characteristics. | | | | | | | | | | | | | | | | |

## Sensitivity analyses

*Without predictors*

In the main paper we adjust in all regressions using variables in Table 1, <5% of which are imputed at the median. To test sensitivity of our key results to imputation (and other potential problems from multivariable regression, e.g. collinearity), we reran the analyses as a univariate regression with weights attached.

We present the results below; they are consistent with the main paper.

RQ1: Association between characteristics and unpaid care hours (N=542)

RQ2: Association between characteristics and time taken off work (N=175)

| **Relationship:** *Same gender* | **Marginal effect** | **95% confidence interval** |
| --- | --- | --- |
| Missed work: Any time | 0.07 | -0.04 to 0.19 |
| Missed work: Before bereavement | 0.07 | -0.06 to 0.21 |
| Missed work: When bereaved | 0.02 | -0.08 to 0.12 |

RQ3: Association between characteristics and healthcare costs (N=542)

| **Relationship:** *Same gender* | **Marginal effect (£)** | **95% confidence interval** |
| --- | --- | --- |
| Healthcare costs: All six months | 45 | -270 to 360 |
| Healthcare costs: Three months prior | -33 | -331 to 265 |
| Healthcare costs: Three months after | **79*** | **2 to 156** |

*p<0.05

*Without propensity score weights*

In the main paper we apply propensity score weights. To test sensitivity of our key results to this choice, we rerun the analyses without weights.

We present the results below; they are consistent with the main paper.

RQ1: Association between characteristics and unpaid care hours (N=542)

RQ2: Association between characteristics and time taken off work (N=175)

| **Relationship:** *Same gender* | **Marginal effect** | **95% confidence interval** |
| --- | --- | --- |
| Missed work: Any time | 0.09 | -0.14 to 0.20 |
| Missed work: Before bereavement | 0.11 | -0.02 to 0.24 |
| Missed work: When bereaved | 0.07 | -0.04 to 0.18 |

RQ3: Association between characteristics and healthcare costs (N=542)

| **Relationship:** *Same gender* | **Marginal effect (£)** | **95% confidence interval** |
| --- | --- | --- |
| Healthcare costs: All six months | 180 | -39 to 397 |
| Healthcare costs: Three months prior | 86 | -108 to 280 |
| Healthcare costs: Three months after | **93**** | **24 to 162** |

*p<0.05, **p<0.01

*Different cost model*

In the main paper we use GLM (gamma, log) to model costs. To test sensitivity of our key results to this choice, we rerun the analyses with GLM (gamma, power 0.5) and OLS on the log-transformed outcome.

We present the results below; they are consistent with the main paper.

RQ3: Association between characteristics and healthcare costs (N=542) using GLM (gamma, power 0.5)

| **Relationship:** *Same gender* | **Marginal effect (£)** | **95% confidence interval** |
| --- | --- | --- |
| Healthcare costs: All six months | 0.44 | -0.03 to 0.92 |
| Healthcare costs: Three months prior | 0.09 | -0.47 to 0.65 |
| Healthcare costs: Three months after | **0.64**** | **0.17 to 1.11** |

*p<0.05, **p<0.01

RQ3: Association between characteristics and log of healthcare costs (N=542) using OLS

| **Relationship:** *Same gender* | **Marginal effect (£)** | **95% confidence interval** |
| --- | --- | --- |
| Healthcare costs: All six months | 180 | -39 to 400 |
| Healthcare costs: Three months prior | 86 | -109 to 281 |
| Healthcare costs: Three months after | **94**** | **25 to 162** |

*p<0.05, **p<0.01

## References

1. Austin PC. Balance diagnostics for comparing the distribution of baseline covariates between treatment groups in propensity-score matched samples. *Stat Med*. Nov 10 2009;28(25):3083-107. doi:10.1002/sim.3697
