# Supplementary material for: Unpaid care, time taken off work and healthcare costs before and after partner bereavement among same-gender and different-gender partners: A national population-based study
Source: Palliat Med. 2025 Aug 16;39(9):977–86. doi: 10.1177/02692163251355796 (PMC12476458; doi:10.1177/02692163251355796)
Supplement: sj-docx-2-pmj-10.1177_02692163251355796 – Supplemental material for Unpaid care, time taken off work and healthcare costs before and after partner bereavement among same-gender and different-gender partners: A national population-based study [file sj-docx-2-pmj-10.1177_02692163251355796.docx]

Study ID: [XXXXX]

**ACCESSCare B: For better care and**

**support in bereavement**

This survey asks for your views and experiences since the recent loss of your partner or spouse. This information will help us to understand the needs of individuals to inform development of services and delivery of care and support in bereavement.

We realise there are a lot of questions to answer, but it would help a great deal if you could try to complete all the pages. We really appreciate you taking the time and trouble to help. Should you require any assistance in completing this survey please do contact us on:

**020 7848 XXXX or** [**XXXXX@kcl.ac.uk**](mailto:XXXXX@kcl.ac.uk).

You may prefer to complete this survey online, if so please enter the web address below, and insert the unique identifier (Study ID) which can be found at the top right hand corner of this page and on the letter that accompanied this survey.

www.XXXX.xxx/xxx

Thank you very much for taking the time to complete this survey.

Some important instructions before you start…

1. Most of the questions can be answered simply by ticking the box next to the answer that best applies. Don't worry if you make a mistake; just cross out the mistake and tick the correct answer.

2. Usually, after answering a question, you should go on to the next one. Sometimes there will be an instruction telling you which question to answer next.

3. Sometimes you are asked to answer in your own words. Please write in the space provided and if necessary use the blank page at the end of the questionnaire.

4. If you cannot remember, do not know, or are unable to answer a particular question, please write that in.

5. The information you give us is **completely confidential** and will not be used in any way that could identify you or your partner or spouse personally.

**Section 1: Your relationship and living situation.** In these first few sections we will be asking you a number of questions about how **you** have been since the loss of your partner or spouse, as well as about the care and support **you** have received. However, it would help us first of all to understand a little bit about you and your partner or spouse, your relationship and living situation.

1. How old are **you**? *Please write down.*

Years old

1. Are **you**…

| Male | 🞎_1_ |
| --- | --- |
| Female | 🞎_2_ |
| Prefer to describe as … | 🞎_3_ |
| Prefer not to say | 🞎_4_ |

1. Does **your** gender identity **differ** from your biological sex at birth?

| Yes | 🞎_1_ |
| --- | --- |
| No | 🞎_2_ |
| Prefer not to say | 🞎_3_ |

1. And, which of the following best describes **your** sexual orientation:

| Completely heterosexual | 🞎_1_ |
| --- | --- |
| Mainly heterosexual  Bisexual | 🞎_2_  🞎_3_ |
| Mainly gay or lesbian | 🞎_4_ |
| Completely gay or lesbian  Other … | 🞎_5_  🞎_6_ |
| Prefer not to say | 🞎_7_ |
| \|  \|  \| \| --- \| --- \|  1. How old was **your partner or spouse** when they died? Please write down.   Years old   1. Was **your partner or spouse**…  \| Male \| 🞎_1_ \| \| --- \| --- \| \| Female \| 🞎_2_ \| \| Preferred to describe as … \| 🞎_3_ \| \| Prefer not to say \| 🞎_4_ \|  1. Did **your partner or spouse’s** gender identity **differ** from their biological sex at birth?  \| Yes \| 🞎_1_ \| \| --- \| --- \| \| No \| 🞎_2_ \| \| Prefer not to say \| 🞎_3_ \|  1. And, which of the following best describes **their** sexual orientation:  \| \| Completely heterosexual \| 🞎_1_ \| \| --- \| --- \| \| Mainly heterosexual  Bisexual \| 🞎_2_  🞎_3_ \| \| Mainly gay or lesbian \| 🞎_4_ \| \| Completely gay or lesbian  Other … \| 🞎_5_  🞎_6_ \| \| Prefer not to say \| 🞎_7_ \| \|  \| \| --- \| --- \| --- \| --- \| --- \| --- \| --- \| --- \| --- \| --- \| --- \| --- \| |  |

|  |  |
| --- | --- |

1. What was your relationship with them? Were you …

| Partner … | 🞎^1^ |  |
| --- | --- | --- |
| Civil Partner … | 🞎^2^ |  |
| Spouse (Husband or Wife) … | 🞎^3^ |  |

1. What was your legal relationship status? Were you …

| Married … | 🞎^1^ |  |
| --- | --- | --- |
| Civil Partnered … | 🞎^2^ |  |
| In a relationship but not married or civil partnered | 🞎^3^ |  |

1. How many years (or part thereof) had you been in a relationship?

| Years |  |  |
| --- | --- | --- |

1. Before they died (or went into a hospital, hospice or nursing home for the last time) was your partner or spouse …

| Living alone … | 🞎^1^ |  |
| --- | --- | --- |
| Living with you only … | 🞎^2^ |  |
| Living with you and others … | 🞎^3^ |  |
| Living with others only …  In a residential or care home ... | 🞎^4^  🞎^5^ |  |

**Section 2: Health and Wellbeing.** In this section we will ask you some general questions about how **you** have been feeling in yourself over the past few weeks. These questions will ask about both your physical and emotional wellbeing.

Have you recently…

|  |  | **Better than usual** | | **Same as usual** | | **Less than usual** | **Much less than usual** |
| --- | --- | --- | --- | --- | --- | --- | --- |
| 1. | Been able to concentrate… | 🞎^1^ | | 🞎^2^ | | 🞎^3^ | 🞎^4^ |
| 2. | Lost much sleep over worry … | 🞎^1^ | | 🞎^2^ | | 🞎^3^ | 🞎^4^ |
| 3. | Felt that you were playing a useful part in things… | 🞎^1^ | | 🞎^2^ | | 🞎^3^ | 🞎^4^ |
| 4. | Felt capable of making decisions… | 🞎^1^ | | 🞎^2^ | | 🞎^3^ | 🞎^4^ |
| 5. | Felt constantly under strain… | 🞎^1^ | | 🞎^2^ | | 🞎^3^ | 🞎^4^ |
| 6. | Felt you could not overcome difficulties… | 🞎^1^ | | 🞎^2^ | | 🞎^3^ | 🞎^4^ |
| 7. | Been able to enjoy normal activities… | 🞎^1^ | | 🞎^2^ | | 🞎^3^ | 🞎^4^ |
| 8. Been able to face up to problems… | | 🞎^1^ | 🞎^2^ | | 🞎^3^ | | 🞎^4^ |
| 9. Been feeling unhappy and depressed… | | 🞎^1^ | 🞎^2^ | | 🞎^3^ | | 🞎^4^ |
| 10. Been losing confidence… | | 🞎^1^ | 🞎^2^ | | 🞎^3^ | | 🞎^4^ |
| 11. Been thinking of yourself as worthless… | | 🞎^1^ | 🞎^2^ | | 🞎^3^ | | 🞎^4^ |
| 12. Been feeling reasonably happy… | | 🞎^1^ | 🞎^2^ | | 🞎^3^ | | 🞎^4^ |

Thinking about your ability to carry out your day-to-day activities, under each heading, please tick the one box that **best describes your health today.**

13. Mobility:

| I have no problems in walking about | 🞎^1^ |
| --- | --- |
| I have slight problems in walking about | 🞎^2^ |
| I have moderate problems in walking about | 🞎^3^ |
| I have severe problems in walking about | 🞎^4^ |
| I am unable to walk about | 🞎^5^ |

1. Self-Care:

| I have no problems in washing or dressing myself | 🞎^1^ |
| --- | --- |
| I have slight problems in washing or dressing myself | 🞎^2^ |
| I have moderate problems in washing or dressing myself | 🞎^3^ |
| I have severe problems in washing or dressing myself | 🞎^4^ |
| I am unable to wash or dress myself | 🞎^5^ |

1. Usual Activities (e.g. work, study, housework, family or leisure activities):

| I have no problems doing my usual activities | 🞎^1^ |
| --- | --- |
| I have slight problems doing my usual activities | 🞎^2^ |
| I have moderate problems doing my usual activities | 🞎^3^ |
| I have severe problems doing my usual activities | 🞎^4^ |
| I am unable to do my usual activities | 🞎^5^ |

1. Pain/Discomfort:

| I have no pain or discomfort | 🞎^1^ |
| --- | --- |
| I have slight pain or discomfort | 🞎^2^ |
| I have moderate pain or discomfort | 🞎^3^ |
| I have severe pain or discomfort | 🞎^4^ |
| I have extreme pain or discomfort | 🞎^5^ |

1. Anxiety/Depression:

| I am not anxious or depressed | 🞎^1^ |
| --- | --- |
| I am slightly anxious or depressed | 🞎^2^ |
| I am moderately anxious or depressed | 🞎^3^ |
| I am severely anxious or depressed | 🞎^4^ |
| I am extremely anxious or depressed | 🞎^5^ |


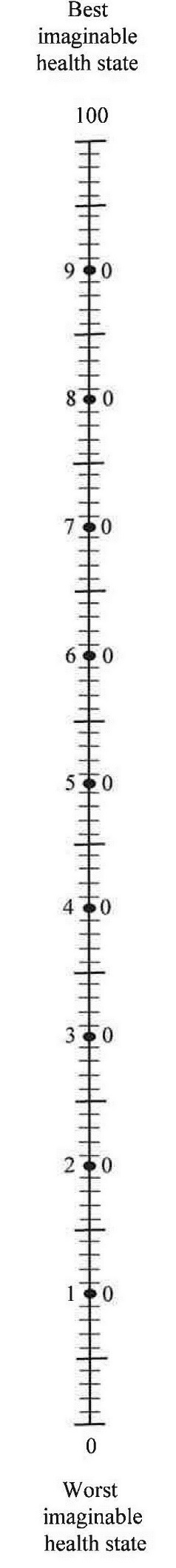


18.

- We would like to know how good or bad your health is **TODAY**.
- This scale is numbered from 0 to 100.
- 100 means the **best** health you can imagine.
- 0 means the  **worst** health you can imagine.
- Mark an X on the scale to indicate how **your** health is **TODAY**.
- Now please write the number you marked on the scale in the box below.

Your Health Today =

| **Section 3: Your day-to-day functioning since your partner or spouse died.** The questions in this section are a little more sensitive. They ask you about how you have been getting on since your partner or spouse died. Please think back to **the time when they died** and answer the items below about your feelings and actions **at that time**.  Please indicate whether each item is **Completely True**, **Mostly True**, **Both True and False**, **Mostly False**, or **Completely False** as it applied to you after they died. Please tick the best answer. |  |  |
| --- | --- | --- |

|  |  | **Completely true** | **Mostly true** | **True & false** | **Mostly false** | **Completely false** |
| --- | --- | --- | --- | --- | --- | --- |
| 1. | After they died I found it hard to get along with certain people … | 🞎^1^ | 🞎^2^ | 🞎^3^ | 🞎^4^ | 🞎^5^ |
| 2. | I found it hard to work well after they died … | 🞎^1^ | 🞎^2^ | 🞎^3^ | 🞎^4^ | 🞎^5^ |
| 3. | After their death I lost interest in my family, friends, and outside activities … | 🞎^1^ | 🞎^2^ | 🞎^3^ | 🞎^4^ | 🞎^5^ |
| 4. | I felt a need to do things that they had wanted to do … | 🞎^1^ | 🞎^2^ | 🞎^3^ | 🞎^4^ | 🞎^5^ |
| 5. | I was unusually irritable after they died … | 🞎^1^ | 🞎^2^ | 🞎^3^ | 🞎^4^ | 🞎^5^ |
| 6. | I couldn't keep up with my normal activities for the first 1-3 months after they died … | 🞎^1^ | 🞎^2^ | 🞎^3^ | 🞎^4^ | 🞎^5^ |
| 7. | I was angry that they left me … | 🞎^1^ | 🞎^2^ | 🞎^3^ | 🞎^4^ | 🞎^5^ |
| 8. | I found it hard to sleep after they died … | 🞎^1^ | 🞎^2^ | 🞎^3^ | 🞎^4^ | 🞎^5^ |
| 9. | I still cry when I think of them … | 🞎^1^ | 🞎^2^ | 🞎^3^ | 🞎^4^ | 🞎^5^ |

Now please answer all the following items about how you **presently** feel about their death.

|  |  | **Completely true** | **Mostly true** | **True & false** | **Mostly false** | **Completely false** |
| --- | --- | --- | --- | --- | --- | --- |
| 10. | I still get upset when I think about their them | 🞎^1^ | 🞎^2^ | 🞎^3^ | 🞎^4^ | 🞎^5^ |
| 11. | I cannot accept their death | 🞎^1^ | 🞎^2^ | 🞎^3^ | 🞎^4^ | 🞎^5^ |
| 12. | Sometimes I very much miss them | 🞎^1^ | 🞎^2^ | 🞎^3^ | 🞎^4^ | 🞎^5^ |
| 13. | Even now it's painful to recall memories of them | 🞎^1^ | 🞎^2^ | 🞎^3^ | 🞎^4^ | 🞎^5^ |
| 14. | I am preoccupied with thoughts (often think) about them | 🞎^1^ | 🞎^2^ | 🞎^3^ | 🞎^4^ | 🞎^5^ |
| 15. | I hide my tears when I think about them | 🞎^1^ | 🞎^2^ | 🞎^3^ | 🞎^4^ | 🞎^5^ |
| 16. | No one will ever take the place in my life of them | 🞎^1^ | 🞎^2^ | 🞎^3^ | 🞎^4^ | 🞎^5^ |
| 17. | I can't avoid thinking about them | 🞎^1^ | 🞎^2^ | 🞎^3^ | 🞎^4^ | 🞎^5^ |
| 18. | I feel it's unfair that they died | 🞎^1^ | 🞎^2^ | 🞎^3^ | 🞎^4^ | 🞎^5^ |
| 19. | Things and people around me still remind me of them | 🞎^1^ | 🞎^2^ | 🞎^3^ | 🞎^4^ | 🞎^5^ |
| 20. | I am unable to accept their death | 🞎^1^ | 🞎^2^ | 🞎^3^ | 🞎^4^ | 🞎^5^ |
| 21. | At times I still feel the need to cry for them | 🞎^1^ | 🞎^2^ | 🞎^3^ | 🞎^4^ | 🞎^5^ |

|  |  |
| --- | --- |

**Section 4: Your current feeling around the loss.** This section focuses on **your current feelings** around the loss of your partner or spouse. These questions are quite sensitive, but please complete them all if you can.

|  |  | | **Never** | | | **Rarely** | | | | | **Sometimes** | | | | **Often** | | | | **Always** | | | | |  |
| --- | --- | --- | --- | --- | --- | --- | --- | --- | --- | --- | --- | --- | --- | --- | --- | --- | --- | --- | --- | --- | --- | --- | --- | --- |
| 1. | I think about this person so much that it’s hard for me to do the things I normally do | | 🞎^1^ | | | 🞎^2^ | | | | | 🞎^3^ | | | | 🞎^4^ | | | | 🞎^5^ | | | | |  |
| 2. | Memories of the person who died upset me | | 🞎^1^ | | | 🞎^2^ | | | | | 🞎^3^ | | | | 🞎^4^ | | | | 🞎^5^ | | | | |  |
| 3. | I feel I cannot accept the death of the person who died | | 🞎^1^ | | | 🞎^2^ | | | | | 🞎^3^ | | | | 🞎^4^ | | | | 🞎^5^ | | | | |  |
| 4. | I feel myself longing for the person who died | | 🞎^1^ | | | 🞎^2^ | | | | | 🞎^3^ | | | | 🞎^4^ | | | | 🞎^5^ | | | | |  |
| 5. | I feel drawn to places and things associated with the person who died | | 🞎^1^ | | | 🞎^2^ | | | | | 🞎^3^ | | | | 🞎^4^ | | | | 🞎^5^ | | | | |  |
| 6. | I can’t help feeling angry about their death | | 🞎^1^ | | | 🞎^2^ | | | | | 🞎^3^ | | | | 🞎^4^ | | | | 🞎^5^ | | | | |  |
| 7. | I feel disbelief over what happened | | 🞎^1^ | | | 🞎^2^ | | | | | 🞎^3^ | | | | 🞎^4^ | | | | 🞎^5^ | | | | |  |
| 8. I feel stunned or dazed over what happened | | | 🞎^1^ | | | 🞎^2^ | | | | 🞎^3^ | | | | | 🞎^4^ | | | | 🞎^5^ | | | | |  |
| 9. Ever since they died it is hard for me to trust people | | | 🞎^1^ | | | 🞎^2^ | | | | 🞎^3^ | | | | | 🞎^4^ | | | | 🞎^5^ | | | | |  |
| 10. Ever since they died I feel like I have lost the ability to care about other people or I feel distant from people I care about | | | 🞎^1^ | | | 🞎^2^ | | | | 🞎^3^ | | | | | 🞎^4^ | | | | 🞎^5^ | | | | |  |
|  | | **Never** | | | | | | **Rarely** | | | | **Sometimes** | | | | | **Often** | | | **Always** | | | | |
| 11. I have pain in the same area of my body or have some of the same symptoms as the person who died | | | | 🞎^1^ | | | | | 🞎^2^ | | | | | 🞎^3^ | | 🞎^4^ | | | | | 🞎^5^ | |  |  |
| 12. I go out of my way to avoid reminders of the person who died | | | | 🞎^1^ | | | | | 🞎^2^ | | | | | 🞎^3^ | | 🞎^4^ | | | | | 🞎^5^ | |  |  |
| 13. I feel that life is empty without the person who died | | | | | 🞎^1^ | | 🞎^2^ | | | | | | 🞎^3^ | | 🞎^4^ | | | 🞎^5^ | | | |  |  |  |
| 14. I hear the voice of the person who died speak to me | | | | | 🞎^1^ | | 🞎^2^ | | | | | | 🞎^3^ | | 🞎^4^ | | | 🞎^5^ | | | |  |  |  |
| 15. I see the person who died stand before me | | | | | 🞎^1^ | | 🞎^2^ | | | | | | 🞎^3^ | | 🞎^4^ | | | 🞎^5^ | | | |  |  |  |
| 16. I feel that it is unfair that I should live when this person died | | | | | 🞎^1^ | | 🞎^2^ | | | | | | 🞎^3^ | | 🞎^4^ | | | 🞎^5^ | | | |  |  |  |
| 17. I feel bitter over this person’s death | | | | | 🞎^1^ | | 🞎^2^ | | | | | | 🞎^3^ | | 🞎^4^ | | | 🞎^5^ | | | |  |  |  |
| 18. I feel envious of others who have not lost someone close | | | | | 🞎^1^ | | 🞎^2^ | | | | | | 🞎^3^ | | 🞎^4^ | | | 🞎^5^ | | | |  |  |  |
| 19. I feel lonely a great deal of the time ever since they died | | | | | 🞎^1^ | | 🞎^2^ | | | | | | 🞎^3^ | | 🞎^4^ | | | 🞎^5^ | | | |  |  |  |

1. Since they died, have you talked to anyone from health and social services, a bereavement service, or a counsellor or therapist about your feelings regarding their illness and death?

| Yes … | 🞎1 |
| --- | --- |
| No … | 🞎2 |

If yes, was this helpful?

| Yes … | 🞎1 |
| --- | --- |
| No … | 🞎2 |

Please use this space to share any more information about your feelings since the loss of your partner or spouse…

___________________________________________________________________________________________________________________________________________________________________________________________________________________________________________________________________________________________________________________________________________________________________________________________________________________________________________________________________________________________________________________________________________________________________________________________________________________

**Section 5: Social networks and support.** The following questions ask you about the people around you now to support you day-to-day. These questions are important in helping us to understand the different types of support individuals may have after the loss of a partner or spouse.

1. About how long does it take for your nearest relative or friend to get to where you live? Think of the time it usually takes door to door.

| Less than 15 minutes | 🞎^1^ |
| --- | --- |
| Between 15 and 30 minutes | 🞎^2^ |
| Between 30 minutes and 1 hour | 🞎^3^ |
| Between 1 and 2 hours | 🞎^4^ |
| Between 2 and 5 hours | 🞎^5^ |
| Between 5 and 12 hours | 🞎^6^ |
| Over 12 hours | 🞎^7^ |

1. People sometimes look to others for companionship, assistance or other types of support. How often is each of the following kinds of support available to you if you need it? Choose one answer for each line.

| **Emotional / Informational Support** | **None of the time** | **A little of the time** | **Some of the time** | **Most of the time** | **All of the time** |
| --- | --- | --- | --- | --- | --- |
| 1. Someone you can count on to listen to you when you need to talk | 🞎^1^ | 🞎^2^ | 🞎^3^ | 🞎^4^ | 🞎^5^ |
| 1. Someone to give you information to help you understand a situation | 🞎^1^ | 🞎^2^ | 🞎^3^ | 🞎^4^ | 🞎^5^ |
| 1. Someone to give you good advice about a crisis | 🞎^1^ | 🞎^2^ | 🞎^3^ | 🞎^4^ | 🞎^5^ |
| 1. Someone to confide in or talk to about yourself or your problems | 🞎^1^ | 🞎^2^ | 🞎^3^ | 🞎^4^ | 🞎^5^ |
| 1. Someone whose advice you really want | 🞎^1^ | 🞎^2^ | 🞎^3^ | 🞎^4^ | 🞎^5^ |
|  | **None of the time** | **A little of the time** | **Some of the time** | **Most of the time** | **All of the time** |
| 1. Someone to share your most private worries and fears with | 🞎^1^ | 🞎^2^ | 🞎^3^ | 🞎^4^ | 🞎^5^ |
| 1. Someone to turn to for suggestions about how to deal with a personal problem | 🞎^1^ | 🞎^2^ | 🞎^3^ | 🞎^4^ | 🞎^5^ |
| 1. Someone who understands your problems | 🞎^1^ | 🞎^2^ | 🞎^3^ | 🞎^4^ | 🞎^5^ |
| **Tangiable Support** | **None of the time** | **A little of the time** | **Some of the time** | **Most of the time** | **All of the time** |
| 1. Someone to help you if you were confined to bed | 🞎^1^ | 🞎^2^ | 🞎^3^ | 🞎^4^ | 🞎^5^ |
| 1. Someone to take you to the doctor if you needed it | 🞎^1^ | 🞎^2^ | 🞎^3^ | 🞎^4^ | 🞎^5^ |
| 1. Someone to prepare your meals if you were unable to do it yourself | 🞎^1^ | 🞎^2^ | 🞎^3^ | 🞎^4^ | 🞎^5^ |
| 1. Someone to help with daily chores if you were sick | 🞎^1^ | 🞎^2^ | 🞎^3^ | 🞎^4^ | 🞎^5^ |
| **Affectionate Support** | **None of the time** | **A little of the time** | **Some of the time** | **Most of the time** | **All of the time** |
| 1. Someone who shows you love and affection | 🞎^1^ | 🞎^2^ | 🞎^3^ | 🞎^4^ | 🞎^5^ |
| 1. Someone to love and make you feel wanted | 🞎^1^ | 🞎^2^ | 🞎^3^ | 🞎^4^ | 🞎^5^ |
| 1. Someone who hugs you | 🞎^1^ | 🞎^2^ | 🞎^3^ | 🞎^4^ | 🞎^5^ |

| **Positive Social Interaction** | **None of the time** | **A little of the time** | **Some of the time** | **Most of the time** | **All of the time** |
| --- | --- | --- | --- | --- | --- |
| 1. Someone to have a good time with | 🞎^1^ | 🞎^2^ | 🞎^3^ | 🞎^4^ | 🞎^5^ |
| 1. Someone to get together with for relaxation | 🞎^1^ | 🞎^2^ | 🞎^3^ | 🞎^4^ | 🞎^5^ |
| 1. Someone to do something enjoyable with | 🞎^1^ | 🞎^2^ | 🞎^3^ | 🞎^4^ | 🞎^5^ |
| 1. Someone to do things with to help you get your mind off things | 🞎^1^ | 🞎^2^ | 🞎^3^ | 🞎^4^ | 🞎^5^ |

1. The next questions are about how you feel about different aspects of your life. For each one, tell me how often you feel that way.

|  | Hardly ever | Some of the time | Often |
| --- | --- | --- | --- |
| 1. First, how often do you feel that you lack companionship? | 🞎^1^ | 🞎^2^ | 🞎^3^ |
| 1. How often do you feel left out? | 🞎^1^ | 🞎^2^ | 🞎^3^ |
| 1. How often do you feel isolated from others? | 🞎^1^ | 🞎^2^ | 🞎^3^ |

Please use this space to share any more detail about the support around you. ______________________________________________________________________________________________________________________________________________________________________________________________________________________________

|  |  |  |  |
| --- | --- | --- | --- |

|  |  |
| --- | --- |
|  |  |

**Section 6: Care and Support.** In order for us to understand your experiences, it is helpful for us to know what care and support you and your partner or spouse had access to.

In the next few sections we will be asking you some specific questions about the care services your **partner or spouse accessed**, as well as any care or support **you** received before their death. There are also some questions about any **informal care and support** your partner received, as well as **your experiences of caring** for your partner**.**

There are also some more sensitive questions in this section about your partner or spouse’s death, and any support **you** may have accessed in bereavement.

1. Did your partner or spouse

stay overnight in… Yes No How many times

(please write down)

| A hospital intensive care unit? | 🞎 | 🞎 | times |  |
| --- | --- | --- | --- | --- |
| Another hospital unit or ward? | 🞎 | 🞎 | times |  |
| A hospice? | 🞎 | 🞎 | times |  |
| A nursing, residential or care home? | 🞎 | 🞎 | times |  |
| Did they visit… |  |  |  |  |
| An Accident & Emergency (A&E) department? | 🞎 | 🞎 | times |  |
| An outpatient clinic? | 🞎 | 🞎 | times |  |

| A day care centre? | 🞎 | 🞎 | times |  |
| --- | --- | --- | --- | --- |
|  |  |  |  |  |
|  |  |  |  |  |
|  |  |  |  |  |
|  |  |  |  |  |

|  |  |  |  |
| --- | --- | --- | --- |

1. Please give details of any other services they used while they was **not in hospital or hospice** during the **last three months** before they died. (Please include face-to-face and telephone contacts by or for their, e.g. by you).

Did they have contact with… Yes No How many times

(please write down)

| GPs or family doctors – face-to-face? (e.g. at home, in the GP practice, nursing home) | 🞎 | 🞎 | times |  |
| --- | --- | --- | --- | --- |
| GPs or family doctors – on the telephone? | 🞎 | 🞎 | times |  |
| A district nurse of community nurse? | 🞎 | 🞎 | times |  |
| Another nurse? (specify) | 🞎 | 🞎 | times |  |
| A palliative care or ‘hospice at home’ team? | 🞎 | 🞎 | times |  |
| A physiotherapist? | 🞎 | 🞎 | times |  |

| An occupational therapist? | 🞎 | 🞎 | times |  |
| --- | --- | --- | --- | --- |
| A psychiatrist? | 🞎 | 🞎 | times |  |
| A psychologist or counsellor? | 🞎 | 🞎 | times |  |
| A spiritual care person / faith leader?  (e.g. a chaplain, rabbi, imam etc.) | 🞎 | 🞎 | times |  |
| A social worker? | 🞎 | 🞎 | times |  |
| Other professionals? (specify) | 🞎 | 🞎 | times |  |

Yes No Usually, how many hours per

week (please write down)

| 1. In the  **last 3 months** before they died, did they have any **paid** help from a home care worker | 🞎 | 🞎 | times |  |
| --- | --- | --- | --- | --- |
| 1. Did they use any **private** care in the **last 3 months** before they died (e.g. staying in a private hospital, getting private nurses at home, doing scans or tests privately)? | 🞎 | 🞎 | times |  |

Please use this space to share any more detail about the types of care your partner or spouse received…

_________________________________________________________________________________________________________________________________________________________________________________________________________________________________________________________________________________________________________

1. Altogether, do you feel that the help and support your partner or spouse, you and those close to you received from **all the services** **at the hospital** was:

| Excellent ……………….………….. | 🞎^1^ |
| --- | --- |
| Very good …………….…………… | 🞎^2^ |
| Good ………………………………….. | 🞎^3^ |
| Fair ……………………………………. | 🞎^4^ |
| Poor ………………………………….. | 🞎^5^ |
| Very poor ……………….…………  Not applicable / did not stay in hospital | 🞎^6^  🞎^7^ |

1. Altogether, do you feel that the help and support your partner or spouse, you and those close to you received from **the services** **at the hospice** was:

| Excellent ……………….………….. | 🞎^1^ |
| --- | --- |
| Very good …………….…………… | 🞎^2^ |
| Good ………………………………….. | 🞎^3^ |
| Fair ……………………………………. | 🞎^4^ |
| Poor ………………………………….. | 🞎^5^ |
| Very poor ……………….…………  Not applicable / did not stay in hospice | 🞎^6^  🞎^7^ |

1. Altogether, do you feel that the help and support your partner or spouse, you and those close to you received from **all the services** **at home** was:

| Excellent ……………….………….. | 🞎^1^ |
| --- | --- |
| Very good …………….…………… | 🞎^2^ |
| Good ………………………………….. | 🞎^3^ |
| Fair ……………………………………. | 🞎^4^ |
| Poor ………………………………….. | 🞎^5^ |
| Very poor ……………….…………  Not applicable / did not receive care at home | 🞎^6^  🞎^7^ |

1. Altogether, do you feel that the help and support your partner or spouse, you and those close to you received from **all the services** **at the nursing, residential or care home** was:

| Excellent ……………….………….. | 🞎^1^ |
| --- | --- |
| Very good …………….…………… | 🞎^2^ |
| Good ………………………………….. | 🞎^3^ |
| Fair ……………………………………. | 🞎^4^ |
| Poor ………………………………….. | 🞎^5^ |
| Very poor ……………….…………  Not applicable / did not stay in nursing, residential or care home | 🞎^6^  🞎^7^ |

|  |  | | Please use this space to share any more detail about the quality of care your partner or spouse received…  ___________________________________________________________________________________________________________________________________________________________________________________________________________________________________________________________________________________________________________________________________________________________________________ |  |
| --- | --- | --- | --- | --- |
|  |  | |  |  |
|  |  | |  |  |
|  |  | |  |  |
|  |  | |  |  |
|  |  | |  |  |
|  |  | |  |  |
|  |  |  |  |  |

| **Section 7: Other sources of care and support.** It is also important for us to understand the other people who may have provided **informal care or support** to your partner or spouse in **last the three months** before they died**.** This includes support that **you, friends, family and other people close to you** may have provided. |  |
| --- | --- |

1. Did **you** look after or help take care of your partner or spouse in the **3 months before they died**?

| Yes … | 🞎^1^ |
| --- | --- |
| No … | 🞎^2^ |

1. Did other friends or family members look after or help take care of them?

| Yes … | 🞎^1^ |
| --- | --- |
| No … | 🞎^2^ |

If yes, how many other friends and family members helped (excluding you)?

| One person | 🞎^1^ |
| --- | --- |
| Two persons | 🞎^2^ |
| Three persons | 🞎^3^ |
| Four persons | 🞎^4^ |
| Five or more persons | 🞎^5^ |

|  |  |
| --- | --- |

This question is important so we can understand how much help people receive from their family and friends. We realise it is not easy to break this down into hours per week. However, it would help a great deal if you could try and remember as best you can. We would like to know on average **how much time you and other friends and family members spent helping your partner or spouse** with particular activities per week **during the last 3 months before they died.**

1. Please give details of any help that you, friends and family members gave to your partner or spouse in the **last 3 months** before they died (this is just family and friends, and those close to you, not paid carers).

Did you and other friends or

family help with… Yes No If yes, usually how many hours

per week (please tick)

| Personal care?  (E.g. washing, dressing) | Yes  🞎 | No 🞎 | \| Less than 5 hours per week \| 🞎^1^ \| \| --- \| --- \| \| 5-9 hours per week \| 🞎^2^ \| \| 10-19 hours per week \| 🞎^3^ \| \| 20-49 hours per week \| 🞎^4^ \| \| 50 or more hours per week \| 🞎^5^ \| |  |
| --- | --- | --- | --- | --- | --- | --- | --- | --- | --- | --- | --- | --- | --- | --- |
| Medical procedures?  (E.g. taking medicines) | Yes  🞎 | No  🞎 | \| Less than 5 hours per week \| 🞎^1^ \| \| --- \| --- \| \| 5-9 hours per week \| 🞎^2^ \| \| 10-19 hours per week \| 🞎^3^ \| \| 20-49 hours per week \| 🞎^4^ \| \| 50 or more hours per week \| 🞎^5^ \| |  |
| Going to appointments or treatments? | Yes  🞎 | No  🞎 | \| Less than 5 hours per week \| 🞎^1^ \| \| --- \| --- \| \| 5-9 hours per week \| 🞎^2^ \| \| 10-19 hours per week \| 🞎^3^ \| \| 20-49 hours per week \| 🞎^4^ \| \| 50 or more hours per week \| 🞎^5^ \| |  |
| Household tasks?  (e.g. shopping, cooking) | Yes  🞎 | No  🞎 | \| Less than 5 hours per week \| 🞎^1^ \| \| --- \| --- \| \| 5-9 hours per week \| 🞎^2^ \| \| 10-19 hours per week \| 🞎^3^ \| \| 20-49 hours per week \| 🞎^4^ \| \| 50 or more hours per week \| 🞎^5^ \| |  |

Did you or other friends or

family help with… Yes No If yes, usually how many hours

per week (please tick)

| Time spent ‘on call’  (i.e. being with their if needed) | Yes  🞎 | No  🞎 | \| Less than 5 hours per week \| 🞎^1^ \| \| --- \| --- \| \| 5-9 hours per week \| 🞎^2^ \| \| 10-19 hours per week \| 🞎^3^ \| \| 20-49 hours per week \| 🞎^4^ \| \| 50 or more hours per week \| 🞎^5^ \| |  |
| --- | --- | --- | --- | --- | --- | --- | --- | --- | --- | --- | --- | --- | --- | --- |
| Time spent with their (e.g. visiting, doing things together) | Yes 🞎 | No 🞎 | \| Less than 5 hours per week \| 🞎^1^ \| \| --- \| --- \| \| 5-9 hours per week \| 🞎^2^ \| \| 10-19 hours per week \| 🞎^3^ \| \| 20-49 hours per week \| 🞎^4^ \| \| 50 or more hours per week \| 🞎^5^ \| |  |

1. Did you stop working or reduce work due to their illness in the **last 3 months** before they died (please include paid or unpaid days off work and any carer’s leave)?

|  |  |  | | |  | |  |
| --- | --- | --- | --- | --- | --- | --- | --- |
| Yes… | | | 🞎1 |  | |  | |
| No, I was retired… | | | 🞎2 |  | |  | |
| No, I was unemployed… | | | 🞎3 |  | |  | |
| No, I was studying… | | | 🞎4 |  | |  | |
| No, I carried on working equal hours... | | | 🞎5 |  | |  | |

If yes, how many days did you have off work? (please write down) days

1. What about in the  **3 months** after they died, did you stop working or reduce work due to the effects of bereavement (please include paid or unpaid days off work and any compassionate leave)?:

|  |  |  | | |  | |  |
| --- | --- | --- | --- | --- | --- | --- | --- |
| Yes … | | | 🞎1 |  | |  | |
| No, I was retired… | | | 🞎2 |  | |  | |
| No, I was unemployed… | | | 🞎3 |  | |  | |
| No, I was studying… | | | 🞎4 |  | |  | |
| No, I carried on working equal hours... | | | 🞎5 |  | |  | |

If yes, how many days did you have off work? (please write down) days

|  |
| --- |
| **Section 8: providing care and support to your partner or spouse.** We would like to ask you about the experience of caring for your partner or spouse in **the last three months of their life**. Please **think back** and answer the items below about **your feelings and actions at that time.**  Please indicate whether each item is **Never**, **Rarely**, **Sometimes**, **Quite frequently** or **Nearly Always** as it applied to you after they died. Please tick the best answer. **Please remember there are no right or wrong answers.** |

| Did you feel… |  |  |  |  |  |  |
| --- | --- | --- | --- | --- | --- | --- |
|  | **Never** | **Rarely** | **Sometimes** | **Quite frequently** | **Nearly always** |  |
| 1. …that because of the time you spent with your partner or spouse you didn’t have enough time for yourself? | _0_ | _1_ | _2_ | _3_ | _4_ |  |
| 1. …stressed between caring for your partner or spouse and trying to meet other responsibilities for your family or work? | _0_ | _1_ | _2_ | _3_ | _4_ |  |
| 1. …angry towards your partner or spouse when you were around their? | _0_ | _1_ | _2_ | _3_ | _4_ |  |
| 1. …that your partner or spouse affected your relationship with other family members or friends in a negative way? | _0_ | _1_ | _2_ | _3_ | _4_ |  |
| 1. …strained when you were around your partner or spouse? | _0_ | _1_ | _2_ | _3_ | _4_ |  |
| 1. …that your health suffered because of your involvement with your partner or spouse ? | _0_ | _1_ | _2_ | _3_ | _4_ |  |

|  | **Never** | **Rarely** | **Sometimes** | **Quite frequently** | **Nearly always** |
| --- | --- | --- | --- | --- | --- |
|  |  |  |  |  |  |
| 1. …that you didn’t have as much privacy as you would like because of your partner or spouse? | _0_ | _1_ | _2_ | _3_ | _4_ |
| 1. …that your social life suffered because you were caring for your partner or spouse? | _0_ | _1_ | _2_ | _3_ | _4_ |
| 1. …that you lost control of your life since your partner or spouse ’s illness? | _0_ | _1_ | _2_ | _3_ | _4_ |
| 1. …uncertain about what to do about your partner or spouse? | _0_ | _1_ | _2_ | _3_ | _4_ |
| 1. …that you should have been doing more for your partner or spouse? | _0_ | _1_ | _2_ | _3_ | _4_ |
| 1. …that you could have done a better job in caring for your partner or spouse? | _0_ | _1_ | _2_ | _3_ | _4_ |
| 1. …happy when you were around your partner or spouse? | _0_ | _1_ | _2_ | _3_ | _4_ |
| 1. …happy to have had the opportunity of caring for their? | _0_ | _1_ | _2_ | _3_ | _4_ |
| 1. …good about your ability as a caregiver? | _0_ | _1_ | _2_ | _3_ | _4_ |
| 1. … that you shared pleasurable experiences with your partner or spouse? | _0_ | _1_ | _2_ | _3_ | _4_ |
| 1. …that caring for your partner or spouse strengthened your relationship? | _0_ | _1_ | _2_ | _3_ | _4_ |
| 1. …that caring for your partner or spouse gave you a sense of purpose or meaning? | _0_ | _1_ | _2_ | _3_ | _4_ |
| 1. …that you have received more emotional support from others since caring for your partner or spouse? | _0_ | _1_ | _2_ | _3_ | _4_ |
| 1. …that you have more control over your life since caring for your partner or spouse? | _0_ | _1_ | _2_ | _3_ | _4_ |
|  | Please use this space to share any more detail about your experience of caring for your partner or spouse…  _________________________________________________________________________________________________________________________________________________________________________________________________________________________________________________________________________________________________________ |  |  |  |  |

|  | **Never** | **Rarely** | **Sometimes** | **Quite frequently** | **Nearly always** |
| --- | --- | --- | --- | --- | --- |
|  |  |  |  |  |  |

**Section 9: Care and Support you received before the death of your partner or spouse.** Please give details of any services (NHS or private) that you used for **your own health** during the **last three months** **before** your partner or spouse died (please included face-to-face and telephone contacts).

|  |  |
| --- | --- |

| 1. Did you have contact with: | | **YES** | **NO** | Approximately how many times,  (please write down) | |  |
| --- | --- | --- | --- | --- | --- | --- |
| GPs or family doctors – face-to-face? | | 🞎 | 🞎 | times | |  |
| GPs or family doctors – on the telephone? | | 🞎 | 🞎 | times | |  |
| A district nurse of community nurse? | | 🞎 | 🞎 | times | |  |
| Another nurse? (specify) | | 🞎 | 🞎 | times | |  |
| A palliative care or ‘hospice at home’ team? | 🞎 | | 🞎 | | times |  |
| A physiotherapist? | 🞎 | | 🞎 | | times |  |

| An occupational therapist? | 🞎 | 🞎 | times |  |
| --- | --- | --- | --- | --- |
| A psychiatrist? | 🞎 | 🞎 | times |  |
| A psychologist or counsellor? | 🞎 | 🞎 | times |  |
| A spiritual care person or faith leader?  (e.g. a chaplain, rabbi, or imam etc.) | 🞎 | 🞎 | times |  |
| A social worker? | 🞎 | 🞎 | times |  |
| Other professionals? (specify) | 🞎 | 🞎 | times |  |

**Section 10: The circumstances of your partner or spouse’s death.** The following questions are a little bit more sensitive. They ask about how long your partner or spouse was ill for, their main problems and concerns, and how expected or unexpected the death was. This information is important to help us to understand how this may affect your experiences as a bereaved partner or spouse.

1. During the last week before they died, what if any were their main problems or concerns (including physical, social, psychological and/or spiritual concerns). Please write down.
2. How long had they been ill before they died?

| A day or more, but less than a week | 🞎^1^ |
| --- | --- |
| One week or more, but less than one month | 🞎^2^ |
| One month or more, but less than six months | 🞎^3^ |
| Six months or more, but less than one year | 🞎^4^ |
| One year or more, but less than three years | 🞎^5^ |
| Three years or more | 🞎^6^ |

1. Did **you** realise that they was going to die because of their illness?

| Yes | 🞎^1^ |
| --- | --- |
| No | 🞎^2^ |

1. If yes, how long before their death did you realise they was going to die?

| A day or more, but less than a week | 🞎^1^ |
| --- | --- |
| One week or more, but less than one month | 🞎^2^ |
| One month or more, but less than six months | 🞎^3^ |
| Six months or more, but less than one year | 🞎^4^ |
| One year or more, but less than three years | 🞎^5^ |
| Three years or more | 🞎^6^ |

1. Did **they** know that they was likely to die because of their illness? *Please tick one only.*

| Yes, they certainly knew | 🞎^1^ |
| --- | --- |
| Yes, they probably knew | 🞎^2^ |
| No, they probably did not know | 🞎^3^ |
| No, they definitely did not know | 🞎^4^ |
| Not sure whether they knew or not | 🞎^5^ |

1. How would you describe their attitude to the illness?

| Definitely accepting | 🞎^1^ |
| --- | --- |
| Fairly accepting | 🞎^2^ |
| Not at all accepting | 🞎^3^ |
| Can’t say | 🞎^4^ |

1. Did any health professional discuss with **them** the fact that they was likely to die because of the illness?

| Yes | 🞎^1^ |
| --- | --- |
| No | 🞎^2^ |
| Don’t know | 🞎^3^ |

1. Did any health professional discuss with **you, other family members or friends** the fact that they was likely to die because of the illness?

| Yes | 🞎^1^ |
| --- | --- |
| No | 🞎^2^ |
| Don’t know | 🞎^3^ |

1. Over the last week before they died, how much information was given to their, you and their family or friends?

| Full information – always felt free to ask what we wanted | 🞎^1^ |
| --- | --- |
| Information given but hard to understand | 🞎^2^ |
| Information given on request but would have liked more | 🞎^3^ |
| Very little given and some questions were avoided | 🞎^4^ |
| None at all | 🞎^5^ |

Please use this space to share any more detail about your experience of communication with healthcare professionals

_________________________________________________________________________________________________________________________________________________________________________________________________________________________________________________________________________________________________________­­­­­­­­­­­­­­­­­­­­­­­­­­­­­­­­­­­­­­­­­­­______________________________________________________________________________________________________________________________________________________________________________________________________

1. Where was your partner or spouse when they died?

| In their own home | 🞎^1^ |
| --- | --- |
| In the home of a relative or friend | 🞎^2^ |
| In a hospice | 🞎^3^ |
| In a hospital | 🞎^4^ |
| In a nursing, residential or care home | 🞎^5^ |
| Elsewhere (specify where): | 🞎^6^ |

1. How long had they been there before they died?

| Less than 24 hours | 🞎^1^ |
| --- | --- |
| A day or more, but less than a week | 🞎^2^ |
| One week or more, but less than one month | 🞎^3^ |
| One month or more, but less than six months | 🞎^4^ |
| Six months or more | 🞎^5^ |

1. As far as you know, where would they have preferred to die?

| In their own home | 🞎^1^ |
| --- | --- |
| In the home of a relative or friend | 🞎^2^ |
| In a hospice | 🞎^3^ |
| In a hospital | 🞎^4^ |
| In a nursing, residential or care home | 🞎^5^ |
| Elsewhere (specify where): | 🞎^6^ |

| They did not have a preference | 🞎^7^ |
| --- | --- |
| Don’t know | 🞎^8^ |

1. Did they discuss their preference with you, other family members or friends?

| Yes | 🞎^1^ |
| --- | --- |
| No | 🞎^2^ |
| Don’t know | 🞎^3^ |

1. Did they discuss their preference with any health professional?

| Yes | 🞎^1^ |
| --- | --- |
| No | 🞎^2^ |
| Don’t know | 🞎^3^ |

1. Were you with their when they died?

| Yes | 🞎^1^ |
| --- | --- |
| No | 🞎^2^ |

1. Were other family members or friends with their when they died?

| Yes | 🞎^1^ |
| --- | --- |
| No | 🞎^2^ |
| Don’t know | 🞎^3^ |

1. Were there any aspects of the care received that they, you, other family members and friends were unhappy with?

| Yes | 🞎^1^ |
| --- | --- |
| No | 🞎^2^ |

Please use this space to share any more detail about the care your partner or spouse received.

_____________________________________________________________________________________________________________________________________________________________________________________________________________________________________________________

1. If yes, was anything done about this? Tick as many as apply.

| Yes, this was discussed with a health professional | 🞎^1^ |
| --- | --- |
| Yes, a formal complaint was made | 🞎^2^ |
| No, no action was taken | 🞎^3^ |

1. Did **you** talk to anyone from health and or social services or from a bereavement service about your feelings regarding their illness and/or anticipated death **before** they died?

| Yes, all the time | 🞎^1^ |
| --- | --- |
| Most of the time | 🞎^2^ |
| Some of the time | 🞎^3^ |
| Occasionally | 🞎^4^ |
| Not very often | 🞎^5^ |
| Not at all | 🞎^6^ |

1. If yes, was this discussion helpful?

| Yes | 🞎^1^ |
| --- | --- |
| No | 🞎^2^ |

Please use this space to share any more detail about your experience of pre-bereavement support, or the support you would have liked to have received. _______________________________________________________________________________________________________________________________________________________________________________________________________________________________________

**Section 11: Care and Support you received after the death of your partner or spouse.** Please give details of any services (NHS or private) that you used **for your own health** during the **first three months after** your partner or spouse died (please included face-to-face and telephone contacts).

| Did you have contact with: | **YES** | **NO** | Approximately how many times,  (please write down) |  |
| --- | --- | --- | --- | --- |
| GPs or family doctors – face-to-face? | 🞎 | 🞎 | times |  |
| GPs or family doctors – on the telephone? | 🞎 | 🞎 | times |  |
| A district nurse of community nurse? | 🞎 | 🞎 | times |  |
| Another nurse? (specify) | 🞎 | 🞎 | times |  |
| A palliative care or ‘hospice at home’ team? | 🞎 | 🞎 | times |  |
| A physiotherapist? | 🞎 | 🞎 | times |  |

| An occupational therapist? | 🞎 | 🞎 | times |  |
| --- | --- | --- | --- | --- |
| A psychiatrist? | 🞎 | 🞎 | times |  |
| A psychologist or counsellor? | 🞎 | 🞎 | times |  |
| A spiritual care person or faith leader?  (e.g. a chaplain, rabbi, and imam etc.) | 🞎 | 🞎 | times |  |
| A social worker? | 🞎 | 🞎 | times |  |
| Other professionals? (specify) | 🞎 | 🞎 | times |  |

**Section 12: Other life experiences.** The questions in the following two sections are quite sensitive. They ask you about **other events** that may have happened to you, or you may have witnessed, in your life. It would be helpful if you could complete these so that we can understand about other significant life events or experiences you may have had.

Listed below are a number of difficult or stressful things that sometimes happen to people. For each event check one or more of the boxes to the right to indicate whether: it happened to you personally; you witnessed it happen to someone else; you’re not sure if it applies to you; or (f) it doesn’t apply to you.

Be sure to consider your entire life (growing up as well as adulthood) as you go through the list of events.

| **Event** | | **Happened to me** | | **Witnessed**  **it** | | | | **Not sure** | **Doesn’t apply** | | |  |
| --- | --- | --- | --- | --- | --- | --- | --- | --- | --- | --- | --- | --- |
| 1. Natural disaster (for example, flood, hurricane, tornado, earthquake) | 🞎^1^ | | | | 🞎^2^ | 🞎^3^ | | | | | 🞎^4^ | |
| 1. Fire or explosion | 🞎^1^ | | | | 🞎^2^ | 🞎^3^ | | | | | 🞎^4^ | |
| 1. Transportation accident (for example, car accident, boat accident, train wreck, plane crash) | 🞎^1^ | | | | 🞎^2^ | 🞎^3^ | | | | | 🞎^4^ | |
| 1. Serious accident at work, home, or during recreational activity | 🞎^1^ | | | | 🞎^2^ | 🞎^3^ | | | | | 🞎^4^ | |
| 1. Exposure to toxic substance (for example, dangerous chemicals, radiation) | 🞎^1^ | | | | 🞎^2^ | 🞎^3^ | | | | | 🞎^4^ | |
| 1. Physical assault (for example, being attacked, hit, slapped, kicked, beaten up) | 🞎^1^ | | | | 🞎^2^ | 🞎^3^ | | | | | 🞎^4^ | |
| **Event** | **Happened**  **to me** | | **Witnessed it** | | | | **Not sure** | | | **Doesn’t apply** | |  |
| 1. Sexual assault (for example, rape, attempted rape, made to perform any type of sexual act through force or threat of harm) | 🞎^1^ | | | | 🞎^2^ | 🞎^3^ | | | | | 🞎^4^ | |
| 1. Other unwanted or uncomfortable sexual experience | 🞎^1^ | | | | 🞎^2^ | 🞎^3^ | | | | | 🞎^4^ | |
| 1. Combat or exposure to a war zone (in the military or as a civilian) | 🞎^1^ | | | | 🞎^2^ | 🞎^3^ | | | | | 🞎^4^ | |
| 1. Captivity (for example, being kidnapped, abducted, held hostage, prisoner of war) | 🞎^1^ | | | | 🞎^2^ | 🞎^3^ | | | | | 🞎^4^ | |
| 1. Life-threatening illness or injury | 🞎^1^ | | | | 🞎^2^ | 🞎^3^ | | | | | 🞎^4^ | |
| 1. Severe human suffering | 🞎^1^ | | | | 🞎^2^ | 🞎^3^ | | | | | 🞎^4^ | |
| 1. Sudden violent death (for example, homicide, suicide) | 🞎^1^ | | | | 🞎^2^ | 🞎^3^ | | | | | 🞎^4^ | |
| 1. Sudden accidental death | 🞎^1^ | | | | 🞎^2^ | 🞎^3^ | | | | | 🞎^4^ | |
| 1. Serious injury, harm, or death you caused to someone else | 🞎^1^ | | | | 🞎^2^ | 🞎^3^ | | | | | 🞎^4^ | |
| 1. Any other very stressful event or experience | 🞎^1^ | | | | 🞎^2^ | 🞎^3^ | | | | | 🞎^4^ | |

**Previous bereavement**

1. When you were a child (under 18), did you experience the death of someone close to you (please tick all that apply):

| Grandparent | 🞎1 |
| --- | --- |
| Parent | 🞎2 |
| Sibling | 🞎3 |
| Other close family member | 🞎4 |
| Close friend | 🞎5 |

1. Before the recent loss of your partner or spouse, since turning 18, have you experienced the death of someone close to you (please tick all that apply):

| Previous partner | 🞎1 |
| --- | --- |
| Child | 🞎2 |
| Parent | 🞎3 |
| Sibling | 🞎4 |
| Grandparent | 🞎5 |
| Other close family member | 🞎6 |
| Close friend | 🞎7 |

Please use this space to share any more detail about your life experiences. ____________________________________________________________________________________________________________________________________________________________________________________________________________________________________________________________________________________________________________________________________________________________________________________________________________________________________

**Section 13: experiences of discrimination.** In this section, we want to understand whether you feel you have been treated differently or discriminated against in the past. It would be helpful if you could complete these so that we can understand any negative experiences you may have had in the past, or concerns you have about the future.

1. In your day-to-day life, how often do any of the following things happen to you currently?

|  | **Almost everyday** | **At least once a week** | | **A few times a month** | | **A few times a year** | **Less than once a year** | | | **Never** |  |
| --- | --- | --- | --- | --- | --- | --- | --- | --- | --- | --- | --- |
| 1. You are treated with less courtesy than other people are | 🞎^1^ | 🞎^2^ | 🞎^3^ | | 🞎^4^ | | | 🞎^5^ | 🞎^6^ | | |
| 1. You are treated with less respect than other people are | 🞎^1^ | 🞎^2^ | 🞎^3^ | | 🞎^4^ | | | 🞎^5^ | 🞎^6^ | | |
| 1. You receive poorer service than other people at restaurants or stores | 🞎^1^ | 🞎^2^ | 🞎^3^ | | 🞎^4^ | | | 🞎^5^ | 🞎^6^ | | |
| 1. People act as if they think you are not smart | 🞎^1^ | 🞎^2^ | 🞎^3^ | | 🞎^4^ | | | 🞎^5^ | 🞎^6^ | | |
| 1. People act as if they are afraid of you | 🞎^1^ | 🞎^2^ | 🞎^3^ | | 🞎^4^ | | | 🞎^5^ | 🞎^6^ | | |
| 1. People act as if they think you are dishonest | 🞎^1^ | 🞎^2^ | 🞎^3^ | | 🞎^4^ | | | 🞎^5^ | 🞎^6^ | | |
| 1. People act as if they are better than you are | 🞎^1^ | 🞎^2^ | 🞎^3^ | | 🞎^4^ | | | 🞎^5^ | 🞎^6^ | | |
| 1. You are called names or insulted | 🞎^1^ | 🞎^2^ | 🞎^3^ | | 🞎^4^ | | | 🞎^5^ | 🞎^6^ | | |
| 1. You are threatened or harassed | 🞎^1^ | 🞎^2^ | 🞎^3^ | | 🞎^4^ | | | 🞎^5^ | 🞎^6^ | | |
| 1. You are treated unfairly by health and social care professionals | 🞎^1^ | 🞎^2^ | 🞎^3^ | | 🞎^4^ | | | 🞎^5^ | 🞎^6^ | | |
| 1. You worry that health or social care professionals **will** treat you unfairly   (in the future) | 🞎^1^ | 🞎^2^ | 🞎^3^ | | 🞎^4^ | | | 🞎^5^ | 🞎^6^ | | |

1. If you have answered ‘a few times a year’ (or more frequently) to any of the above, what do you think is the main reason for these experiences?

| Your ancestry or national origins | 🞎^1^ |
| --- | --- |
| Your gender | 🞎^2^ |
| Your race or ethnicity | 🞎^3^ |
| Your age | 🞎^4^ |
| Your religion | 🞎^5^ |
| Your height | 🞎^6^ |
| Your weight | 🞎^7^ |
| Some other aspect of your physical appearance | 🞎^8^ |
| Your sexual orientation | 🞎^9^ |
| Your gender history or gender identity | 🞎^10^ |
| Your education or income level | 🞎^11^ |

1. In general, how comfortable do you feel sharing **your** sexual orientation with other people:

| Very comfortable, I would not hesitate to share this information with others | 🞎_1_ |
| --- | --- |
| Comfortable, I would rarely conceal this information | 🞎_2_ |
| Fairly comfortable, I would share this information if I felt it was relevant | 🞎_3_ |
| Fairly uncomfortable, I would only share this information if I felt it was necessary | 🞎_4_ |
| Uncomfortable, I would rarely share this information | 🞎_5_ |
| Very uncomfortable, I would not share this information with others | 🞎_6_ |

Please use this space to share any more detail about your experiences of discrimination. ______________________________________________________________________________________________________________________________________________________________________________________________________________________________________________________________________________________________________________________________________________________________

**Section 14: Final questions and experiences of survey.** These last few questions are very important, as they tell us a little bit more about you and your partner or spouse, and also how you found the experience of taking part in this study. This information will be used to improve future research studies.

1. Do **you** work in paid or unpaid employment?

| Yes, I work in paid employment | 🞎^1^ |
| --- | --- |
| Yes, I work in unpaid employment | 🞎^2^ |
| No, I do not currently work in paid or unpaid employment | 🞎^3^ |
| No, I am retired from working | 🞎^4^ |

1. Which of the following descriptions comes closest to how **you** feel about **your** household income nowadays?

| Living comfortably on present income | 🞎^1^ |
| --- | --- |
| Coping on present income | 🞎^2^ |
| Difficult on present income | 🞎^3^ |
| Very difficult on present income | 🞎^4^ |
| Don’t know | 🞎^5^ |
| Prefer not to say | 🞎^6^ |

1. What best describes **your** educational level?

| Did not go to school | 🞎^1^ |
| --- | --- |
| Secondary school (e.g. GCSE or O-Levels) | 🞎^2^ |
| Secondary school (e.g. A-Levels) | 🞎^3^ |
| Post secondary school vocational qualification | 🞎^4^ |
| University | 🞎^5^ |

1. Can you tell us, what is **your** religion?

| No religion | 🞎^1^ |  |  |
| --- | --- | --- | --- |
| Christian (including Church of England, Catholic, Protestant and other Christian denominations) | 🞎^2^ |  |  |
| Buddhist | 🞎^3^ |  |  |
| Hindu | 🞎^4^ |  |  |
| Jewish | 🞎^5^ |  |  |
| Muslim | 🞎^6^ |  |  |
| Sikh | 🞎^7^ |  |  |
| Other (please specify) | 🞎^8^ |  |  |
| Prefer not to say | 🞎^9^ |  |  |
| 1. And which ethnic group do **you** belong to?  \| White British \| 🞎^1^ \| \| --- \| --- \| \| White other (specify below) \| 🞎^2^ \| \| Black Caribbean \| 🞎^3^ \| \| Black African \| 🞎^4^ \| \| Black other (specify below) \| 🞎^5^ \| \| Indian \| 🞎^6^ \| \| Pakistani \| 🞎^7^ \| \| Bangladesh \| 🞎^8^ \| \| Chinese \| 🞎^9^ \| \| Other ethnic group (please specify) \| 🞎^10^ \| \| Prefer not to say \| 🞎^11^ \| | | |  |

1. And what was **your partner or spouse’s** religion?

| No religion | 🞎^1^ |
| --- | --- |
| Christian (including Church of England, Catholic, Protestant and other Christian denominations) | 🞎^2^ |
| Buddhist | 🞎^3^ |
| Hindu | 🞎^4^ |
| Jewish | 🞎^5^ |
| Muslim | 🞎^6^ |
| Sikh | 🞎^7^ |
| Other | 🞎^8^ |
| Prefer not to say | 🞎^9^ |

1. And what ethnic group did **your partner of spouse** belong to?

| White British | 🞎^1^ |
| --- | --- |
| White other (specify below) | 🞎^2^ |
| Black Caribbean | 🞎^3^ |
| Black African | 🞎^4^ |
| Black other (specify below) | 🞎^5^ |
| Indian | 🞎^6^ |
| Pakistani | 🞎^7^ |
| Bangladesh | 🞎^8^ |
| Chinese | 🞎^9^ |
| Other ethnic group | 🞎^10^ |
| Prefer not to say | 🞎^11^ |

1. Were there aspects of this survey that you found upsetting?

| Yes | 🞎^1^ |
| --- | --- |
| No | 🞎^2^ |

1. Did you find completing this survey helpful?

| Yes | 🞎^1^ |
| --- | --- |
| No | 🞎^2^ |

Please use this space to share any more detail about your experiences of taking part in this study. _________________________________________________________________________________________________________________________________________________________________________________________________________________________________________________________________________________________________________________________________________________________________________________________________

1. Are you willing to be contacted by the research team to talk more about your experiences?

| Yes | 🞎^1^ |
| --- | --- |
| No | 🞎^2^ |

1. Do you want to receive a brief summary of the findings of this study?

| Yes | 🞎^1^ |
| --- | --- |
| No | 🞎^2^ |

If you have answered yes to either of the previous two questions, please provide us with your preferred contact details:

Name:

Address:

Telephone:

Email:

Thank you for taking the time to complete the survey.

Please use this space to share any more details or expand on any of your previous answers. ______________________________________________________________________________________________________________________________________________________________________________________________________________________________________________________________________________________________________________________________________________________________________________________________________________________________________________________________________________________________________________________________________________________________________________________________________________________________________________________
